# An anti-inflammatory phenotype in visceral adipose tissue of old lean mice, augmented by exercise

**DOI:** 10.1038/s41598-019-48587-2

**Published:** 2019-08-19

**Authors:** A. K. Ziegler, A. Damgaard, A. L. Mackey, P. Schjerling, P. Magnusson, A. T. Olesen, M. Kjaer, C. Scheele

**Affiliations:** 1 Institute of Sports Medicine Copenhagen, Department of Orthopedic Surgery M, Bispebjerg Hospital and Center for Healthy Aging, Faculty of Health and Medical Sciences, University of Copenhagen, Copenhagen, Denmark; 2 Center for Healthy Aging, Department of Biomedical Sciences, Faculty of Healthy and Medical Sciences, University of Copenhagen, Copenhagen, Denmark; 3 Department of Physical Therapy, Musculoskeletal Rehabilitation Research Unit, Bispebjerg Hospital, Copenhagen, Denmark; 4 The Centre of Inflammation and Metabolism and Centre for Physical Activity Research Rigshospitalet, University Hospital of Copenhagen, Copenhagen, Denmark; 5 Novo Nordisk Foundation Center for Basic Metabolic Research, Faculty of Health and Medical Sciences, University of Copenhagen, Copenhagen, Denmark

**Keywords:** Chronic inflammation, Ageing, Fat metabolism

## Abstract

Visceral adipose tissue is an immunogenic tissue, which turns detrimental during obesity by activation of proinflammatory macrophages. During aging, chronic inflammation increases proportional to visceral adipose tissue (VAT) mass and associates with escalating morbidity and mortality. Here, we utilize a mouse model to investigate the inflammatory status of visceral adipose tissue in lean aging mice and assess the effects of exercise training interventions. We randomized adult (11 months; n = 21) and old (23 months; n = 27) mice to resistance training (RT) or endurance training (ET), or to a sedentary control group (S). Strikingly, we observed an anti-inflammatory phenotype in the old mice, consisting of higher accumulation of M2 macrophages and IL-10 expression, compared to the adult mice. In concordance, old mice also had less VAT mass and smaller adipocytes compared to adult mice. In both age groups, exercise training enhanced the anti-inflammatory phenotype and increased PGC1-α mRNA expression. Intriguingly, the brown adipose tissue marker UCP1 was modestly higher in old mice, while remained unchanged by the intervention. In conclusion, in the absence of obesity, visceral adipose tissue possesses a pronounced anti-inflammatory phenotype during aging which is further enhanced by exercise.

## Introduction

Adipose tissue is host to various immune cells and it is well established that during obesity, the amount of inflammatory macrophages increase in adipose tissue^1,2^. Visceral adipose tissue (VAT), surrounding the inner organs, has been shown to be more inflammatory active than subcutaneous adipose tissue (SAT), as increased amounts of visceral/abdominal fat associates with high levels of circulating inflammatory markers^3–6^, and a high number of pro-inflammatory cells in their adipose tissue^1,2,7^. Moreover, due to its anatomical location, VAT directly supplies the liver with venous blood via the portal vein, rendering a prominent role in directing whole body metabolism. Thus, it is possible that visceral fat is the source of circulating low grade inflammation, which might be important for the development of life-style related chronic diseases^8–10^.

Interestingly, in human and rodent studies, ageing is associated with an increase in the amount of visceral adipose tissue and/or level of inflammation^11–15^. It is, however, unclear to what extent these age-related changes are a result of ageing per se or rather the result of changes in life-style with e.g. reduced levels of physical activity without a corresponding reduction in caloric intake. A human cross sectional study reported that whereas ageing is associated with increased inflammation, life-long endurance training resulted in lower circulating levels of inflammatory markers in both young and elderly individuals^16^. Although endurance exercise has been demonstrated to counteract pathological changes in visceral adipose tissue by reducing the amount of total and visceral adipose tissue under conditions of excess fat^17,18^, the effects of exercise training on VAT during ageing under lean conditions currently remains elusive. Indeed, it has been shown that acute endurance exercise can result in an upregulation of the brown fat marker UCP-1 in mice, suggesting a shift of the visceral fat towards a more metabolic active profile (WAT “browning”), but whether this pertains to long term endurance training remains unknown^19^. Indicators of WAT browning, besides UCP-1 upregulation, include increase in mitochondrial enzymes and involvement of macrophages^20–22^. Finally, the mode of exercise might be of importance as some human studies show that endurance but not strength training can reduce the amount of adipose tissue and thereby inflammation^23^.

In the current study, we wanted to investigate the inflammatory status and tissue integrity of VAT in an exercise-training model of lean adult and old mice. The model consisted of adult and old mice that underwent either voluntary RT or voluntary ET treadmill running, followed by examination of visceral (epididymal) fat both with regards to size/structure, markers of inflammation, oxidative capacity, browning, and fibrosis. We hypothesized, that ageing would be related to increased visceral fat mass, larger adipocytes, higher inflammatory activity and lower oxidative capacity, and that regular physical training of different types would counteract these age-related changes and “rejuvenate” visceral adipose cells.

## Methods

### Exercise protocol

Experiments were conducted in accordance with Danish guidelines (Amendment #1306 of November 23, 2007) as approved by the Danish Animal Inspectorate, Ministry of Justice (permit #2014-15-0201-00326).

C57BL/6 male mice (Janvier labs), aged 11 months (Adult) and 23 months (Old), were randomized to either sedentary (S), Endurance training (ET) or Resistance training (RT) intervention. For characteristics and randomization see Table 1. The mice were individually housed with ad libitum access to standard chow and tap water and a 12:12 day/night cycle. The training interventions consisted of 10 weeks voluntary wheel running in custom build wheels (Trixie, 12 cm diameter) with adjustable resistance. The running wheels were available to the mice day and night. The resistance was determined as the weight required to maintain motion in the wheel. In the ET wheels the resistance was fixed at 1.5 g throughout the intervention. In the RT wheels the resistance was 5 g in week 1, 6 g in week 2, and then increased 1 g every second week ending at 10 g in week 9–10 (Fig. s1). Voluntary running in wheels with high resistance has previously been reported to induce a hypertrophic response in contrast to low resistance wheels^24^. Therefore, RT represented strength/resistance training and ET endurance training in the present study. Running wheel activity was continuously monitored by recording wheel turning speed with a custom build sensor system, recorded via by Arduino boards (Arduino Uno and Ethernet SD Shield), and analyzed in MatLab. After the intervention, the mice were euthanized with cervical dislocation and visceral fat pads from the epididymal region were carefully dissected^25^. The mice were resting 3–5 hours prior to sacrifice. All tissue was weighed, right-hand-side tissue was snap-frozen in liquid nitrogen for mRNA analysis, and the left-hand-side tissue was submerged in formalin for subsequent paraffin embedding and immunohistochemistry. Until further analysis, samples were stored at −80 °C or 5 °C respectively.

**Table 1:** Mice randomization and characteristics.

		Adult (n = 21)	Old (n = 27)
Intervention	S	7	10 (3^†^)
(n)	RT	7	6 (1^†^)
	ET	7	6 (1^†^)
	Total	21	22 (5^†^)
Total bodyweight	S	33.2 ± 0.7	33.3 ± 1.3
(g)	RT	33.7 ± 1.0	32.1 ± 0.9
(mean ± SE)	ET	31.8 ± 0.8	30.4 ± 1.24
	Total	32.9 ± 0.5	32.3 ± 0.7
Epididymal fat mass^a,i^	S	592 [433–810]	322 [156–662]
(mg)	RT	538 [447–648]	139 [80–241]
Geometric mean + 95% CI	ET^*^	397 [319–493]	85 [60–121]
	Total	509 [395–637]	182 [91–268]
Adipocyte area^a,i^	S	2892 [2345–3567]	1821 [1171–2831]
(µm^2^)	RT	2712 [2215–3321]	1163 [764–1077]
Geometric mean + 95% CI	ET^*^	2266 [1863–2756]	907 [931–1451]
	Total	2623 [2131–3196]	1297 [941–1642]
CD206 area staining^a,i^	S	0.2 [0.1–0.4]	0.3 [0.2–0.5]
(%)	RT	0.2 [0.1–0.3]	0.7 [0.4–1.3]
Geometric mean + 95% CI	ET^*^	0.4 [0.3–0.5]	1.3 [0.8–2]
	Total	0.3 [0.2–1.4]	0.8 [0.4–2.4]

Randomization and characteristics of included mice: S = Sedentary, ET = Endurance training, RT = Resistance training. ^†^Died prematurely. 95 % CI = 95 % confidence interval. *Different from S, ^a^main effect of time, ^i^main effect of intervention.

### Immunohistochemistry and image analysis

The tissue samples were embedded in paraffin and cut into sections. Samples were stored at 5 °C until staining procedure. Sections were first exposed to xylene 2 × 5 minutes, followed by dehydration with graded alcohol. Samples were then washed 3 times in distilled water, and a hydrophobic barrier pen was used to encircle the sections. Samples were subsequently fixed in Histofix (Histolab Products AB, Askim, Sweden) for 7 minutes. Samples were washed in 0.05 M TBS (500 ml wash buffer, 0.6 mol/l Tris-base, 1.54 mol/l NaCl, ph 7.4–7.6, RegionH, Apoteket, in 4.5 L distilled water) and antigen retrieval was performed by heating samples in 10 mM sodium-citrate pH 6.5 in a water bath. The initial temperature was 65 °C and was subsequently raised to 92 °C at which temperature samples were treated for 12–13 minutes. Afterwards samples were allowed to cool to room temperature and washed in TBS. From this point, 2 protocols were used to stain for either (1) perilipin or (2) CD206.

1. A MOM (Mouse On Mouse) IgG blocker (Vector Laboratories, CAT#MKB-2213) was applied for 1 hour. Samples were incubated with a perilipin primary antibody (Perilipin A, Abcam ab3526 rabbit) diluted 1:100 in MOM blocker and left over night at 4 °C. The following day samples were washed in TBS and incubated with the secondary antibody (Alexa Fluor goat anti-rabbit 568, A-11036, ThermoFisher, 1:500) for 60 minutes 1% BSA in TBS. Samples were washed in TBS and subsequently mounted in Prolong Gold Antifade (Molecular Probes ProLong Gold anti-fade reagent, cat. no. P36935) with DAPI (4′,6-diamidino-2-phenylindole). Samples were then stored dry in the dark for 2 days and stored at −20 °C hereafter.
2. Endogenous peroxidase was quenched using 3% peroxide in TBS for 10 minutes. Samples were then subjected to blocking using 5% goat serum, 2% BSA in TBS for 1 hour and incubated overnight at 4 °C with the primary antibody (rabbit anti-CD206, ab64693) at a 1:16000 dilution in blocking buffer. The following day, all sections were incubated with the secondary antibody (ImmPRESS Reagent anti-rabbit, vector MP-7401) for 30 minutes. Then ImmPACT DAB Peroxidsase Substrate Kit (Vector, SK-4105) was applied for approximately 7 minutes, rendering sites of CD206 staining brown. Samples were then submerged in hematoxylin for 30 seconds and rinsed for 5 minutes in tap water. Finally, samples were mounted in aquamount (Merck, Germany).

Images of both stainings were captured with a BX51 Olympus microscope, using an Olympus DP71 camera with a x20 objective and Cell^F imaging software (Olympus soft Imaging Solutions, Münster, Germany). The fluorescent images of the perilipin-stained tissue were blinded, so that the investigator was blinded to age- and training groups. Using ImageJ (Fiji, ImageJ 1.49) 200 adipocytes were counted and the size of the area they covered was measured. Area with damaged adipocytes cells or non-AT was excluded from the analysis. Finally, we calculated the average adipocyte size in µm^2^.

Bright-field images of the CD206-stained tissue were acquired by blinded assessor. 4 representative snapshots were taken from each sample from the top, bottom, left and right of the section. Image analysis was conducted using ImageJ software (Fiji, ImageJ 1.49). ROI’s of non-adipose tissue were manually removed so that only adipose tissue was visualized. Great care was also taken to remove blood vessels from the image analysis. Afterwards the snapshots were converted to 8-bit grayscale and pixel intensity thresholding was performed. In order to make sure that the all images were thresholded with the same relative intensity, the lowest possible pixel value for CD206 staining was selected. The upper thresholding border was determined as the addition of 100-pixel intensity values to the lowest detectable value. The area covered by CD206 was then calculated as measured stained area pr. total adipose tissue area. This fraction (%) was then used for subsequent statistical analysis.

### RNA extraction and gene expression analysis

Total RNA was extracted from visceral fat samples using TRIzol Reagent (ThermoFisher Scientific) according to manufacturer’s instructions and was quantified using Nanodrop1000 (Thermofisher, USA), First strand cDNA synthesis was performed using 200 ng of total RNA and the High Capacity cDNA Reverse Transcription Kit (Life technologies) according to the manufacturer’s protocol. cDNA was amplified using the SYBR® Green PCR Master Mix (Life technologies) with 300 nM final primer concentration. Melting point analysis for each reaction was done, confirming primer specificity. Quantitative real-time PCR that was carried out in a ViiA 7 real time PCR system (Applied Biosystems). Standard curves were made with diluted cDNA and used for calculation of Ct values. 3 replicates were run and the 2 values closest together were chosen for further analysis. GAPDH was chosen as reference gene. For primers used, see Table s1.

### Statistics

Data were analyzed by using SigmaPlot 13.0 for Windows (Systat Software, San Jose, CA USA). All data were analyzed using 2-way ANOVA to deduct a possible effect of age and intervention. Whenever significant effects were found a Holm Sidak post hoc analysis was performed. The level of statistical significance was set to p < 0.05. All normal distributed data are given as arithmetic mean ± SE unless otherwise stated. However, to obtain Gaussian distributed values, visceral fat mass, adipocyte size, CD206 area fraction and all mRNA analysis were log transformed. All log transformed data are given as geometric mean with back-transformed SE seen in corresponding figures or 95% CI (see Table 1). mRNA results are shown as relative change, compared to the AS group.

## Results

### Training protocol

On average during the intervention, a main effect of age was seen with the old mice running for shorter time and distance, slower, and with significantly less external work done, compared to the adult mice (all p < 0.01, see Table 2). The ET groups ran for longer distance compared to RT and a trend were seen for ET mice to have longer training time (duration) and velocity compared to RT (p = 0.06 and 0.09 respectively). Further, exercised mice exhibited nocturnal activity habits, with 93% of daily running activity taking place at night between 6 PM and 5 AM with no difference between young and old mice.

**Table 2:** Training intervention.

		Adult	Old
Running distance^a^	RT	4.8 ± 0.6	1.8 ± 0.9
km/day	ET^$^	6.6 ± 0.6	2.8 ± 0.7
(mean ± SE):	Total	5.7 ± 0.5	2.3 ± 0.6
Speed^a^	RT	0.34 ± 0.02	0.24 ± 0.04
(m/s)	ET	0.39 ± 0.02	0.30 ± 0.04
(mean ± SE):	Total	0.37 ± 0.01	0.27 ± 0.03
Time^a^	RT	218 ± 23	94 ± 35
(min)	ET	278 ± 13	130 ± 30
(mean ± SE):	Total	248 ± 15	112 ± 22
External work^a^	RT	0.35 ± 0.05	0.13 ± 0.07
(Nm)	ET^$^	0.06 ± 0.01	0.03 ± 0.01
(mean ± SE):	Total	0.21 ± 0.05	0.08 ± 0.04

Training intervention data. ET = Endurance training, RT = Resistance training. ^$^Different from RT, ^a^main effect of time.

### Visceral adipocyte size and fat mass was reduced with age and exercise training in lean mice

**Figure 1:**
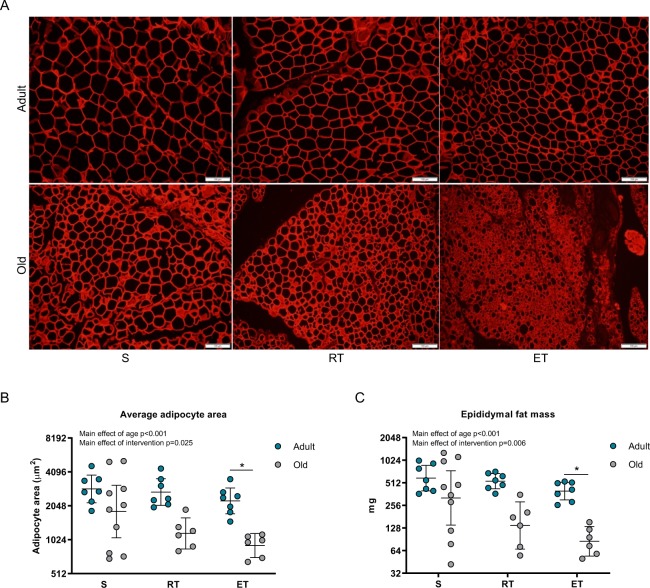
Characteristics of visceral adipose tissue in adult and old mice following exercise training interventions. We characterized the epididymal adipose depot of adult and old mice that were sedentary (S) or had performed either resistance training (RT) or endurance training (ET). (**A)** Representative pictures of perilipin-stained epididymal adipose of adult (top) and old (bottom) mice divided by intervention, 20x objective. (**B)** Average adipocyte area (µm2) measured from 200 adipocytes, based on the perilipin staining. (**C)** Total weight of epididymal adipose depot (mg). (**B**,**C)** Geometric mean ± SE. (**B**,**C)** Y-axis given as log2. ^*^Significantly different from S. Scalebar: 100 µm. Adult S (AS) n = 7, Adult RT (ART) n = 7, Adult ET (AET) n = 7, Old S (OS) n = 10 Old RT (ORT) n = 6; Old ET (OET) n = 6.

To investigate the characteristics of visceral adipose tissue in lean mice during aging, we compared adult (11 months; n = 21) and old (23 months; n = 27) mice. We wanted to assess the effect of exercise training on visceral adipose tissue and thus randomized the mice to resistance training (RT), endurance training (ET) or to a sedentary control group (S). Interestingly, and in contrast to previous observations that visceral adiposity increase with age^12^, we found that the old mice in general exhibited markedly smaller adipocytes (p < 0.001) and less epidydimal fat mass (p < 0.001) compared to the adult mice (Table 1, Fig. 1A–C). This was despite no differences in total body weight (Table 1). Moreover, we found an overall effect of exercise training both in regard to epididymal fat mass (p = 0.006) and adipocyte size (p = 0.025), but with no significant interaction when comparing adult and old mice. In both cases, these differences were driven by the ET groups (p < 0.05; p < 0.05). The differences in visceral adipose tissue phenotype between old and adult mice seemed more pronounced in old mice following endurance exercise training, possibly suggesting higher lipolytic response in the visceral adipose tissue of the old mice compared to the adult. Interestingly, previous reports have discussed the relation between local lipid fluxes and adipose tissue macrophage (ATM) accumulation^26,27^.

### An anti-inflammatory phenotype in visceral adipose tissue of old mice

**Figure 2:**
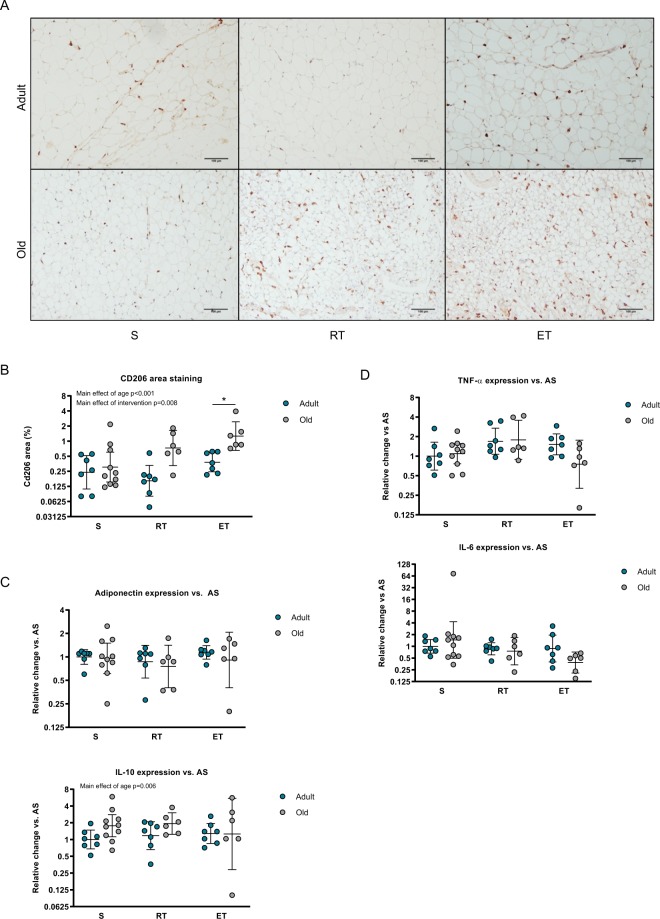
Immunogenic phenotype of visceral adipose tissue in adult and old mice following exercise training interventions. Epididymal adipose from the groups depicted in Fig. 1 were characterized for anti- and pro-inflammatory markers (**A**) Representative images showing CD206 enzymatic staining (brown) using bright field microscopy, 20x objective. (**B**) Quantification of the percentage of total area staining positive for CD206 from 4 snapshots from each mouse. (**C**) Relative gene expression levels of the anti-inflammatory markers, IL-10 and adiponectin. (**D**) Relative gene expression levels of the pro-inflammatory markers, TNF-α and IL-6. Y-axis for all figures given as log2. Data given as geometric mean ± SE. *Significantly different from S. AS n = 7, AET n = 7, ART n = 7, OS n = 10, OET n = 6, ORT n = 6. Scalebar: 100 µm.

To address potential differences in ATM accumulation between adult and old mice, we stained tissue sections for the ATM marker CD206, which is described as a reliable marker of alternatively activated macrophages (M2)^28,29^. We observed an overall effect of age, with the old mice having more positive CD206 staining area than adult (p < 0.001, Fig. 2A,B, Table 1). In concordance with the epididymal fat mass and adipocyte size, there was an overall effect of exercise training (p = 0.008), which seemed to be driven by the ET intervention (p < 0.05). However, we here found an opposing regulation pattern with an increased area positive for CD206 staining in the old vs. adult mice and comparing ET to S (p = 0.007, Fig. 2A,B, Table 1). A trend for an interaction was observed with old mice eliciting a relatively higher CD206 area staining in response to exercise intervention compared to adult mice (both RT and ET, p = 0.077). Furthermore, a trend for significantly more CD206 staining in ET vs. RT was found for both old and adult mice (p = 0.055).

To further address the inflammatory status of the visceral adipose tissue samples, we measured the relative gene expression levels of anti-inflammatory and pro-inflammatory markers. In support of the strongly positive staining for CD206 in the old mice, we detected a higher expression of the anti-inflammatory marker IL-10 in this group (p = 0.006), whereas adiponectin gene expression remained unchanged (Fig. 2C). The proinflammatory markers TNF-α and IL-6 remained unchanged, further emphasizing that the accumulated ATM’s in the old trained mice were not M1 activated (Fig. 2D). In fact, opposite of what would be expected, there was even a trend for interaction of TNF-α, with adult trained mice exhibiting more TNF-α expression compared to trained old mice (Fig. 2D, p = 0.059).

### Markers of fibrosis were not different between groups

**Figure 3:**
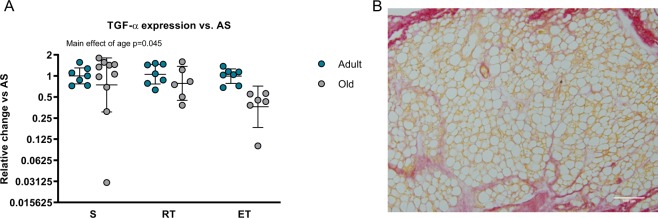
Markers of fibrosis. (**A**) Gene-expression of fibrosis marker TGF-β1 (relative change) in epididymal adipose tissue compared to AS (AS, n = 7, ART, n = 7, AET, n = 7, OS, n = 10, ORT, n = 6, OET, n = 6). Data given as geometric mean ± SE. Y-axis given as log2. (**B)** Picrosirius red staining of VAT in old sedentary mouse revealed no apparent fibrosis in between adipocytes. Brightfield, 20x objective. Scalebar 100 µm.

Increased amounts of M2 macrophages, have been found by other studies to be a hallmark of adipose tissue, peritoneal and pancreatic fibrosis^30–32^. Increased TGF-beta is thought to be a central mediator in this process^32,33^. We therefore measured the gene expression of TGF-β1. Intriguingly, we observed a lower expression in old mice compared to adult mice (p = 0.045) (Fig. 3A). However, from the histology analysis in Figs 1A and 2A, we did observe that the visceral adipose tissue from old mice appeared disorganized, with adipocytes varying greatly in shape and size. To further evaluate whether this phenotype could be related to increased fibrosis, despite not indicated by the TGF-β1 gene expression levels, we applied an exploratory picrosirius red staining on the tissue section of one old mouse, to detect any apparent fibrotic connective tissue in the visceral adipose tissue. However, no histological signs of fibrosis were observed (Fig. 3B). Thus, we do not think that the reduced size of visceral adipose tissue and adipocytes in old mice is due to development of classic fibrosis.

### An oxidative phenotype in VAT promoted by exercise training in old and adult mice

**Figure 4:**
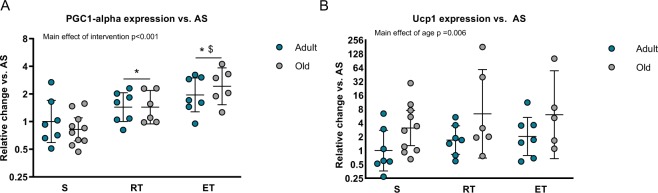
Oxidative markers. Relative gene expression levels in the epididymal adipose tissue from the groups depicted in Fig. 1 were assessed using qPCR. (**A**) Relative gene expression levels of PPARGC-1α, a marker for oxidative capacity (AS, n = 7, ART, n = 7, AET, n = 7, OS, n = 10, ORT, n = 6, OET, n = 6). (**B)** Relative gene expression levels of UCP-1, a marker for browning (AS, n = 7, ART, n = 7, AET, n = 7, OS, n = 10, ORT, n = 6, OET, n = 5). Data given as geometric mean ± SE. *Significantly different from S. ^$^Significantly different from RT. Y-axis given as log2 for both figures.

To further characterize the visceral adipose tissue from the different groups, we measured the relative gene expression of the master regulator of mitochondria, Pgc-1α, as a marker of oxidative capacity. We found an overall response to exercise training (p < 0.001) with the most pronounced effect of ET (p < 0.001 compared to S, p = 0.023 compared to RT) and a smaller effect of RT (p = 0.017) compared to S) (Fig. 4A). Pgc-1α is a co-transcription factor of the brown fat transcriptional program^34^, and browning of white adipose tissue in response to exercise training has been previously reported to occur in mice^19,20,35^. These observations raised the idea that the disorganization and variation of adipocyte size might be related to adipose tissue browning. Therefore, we measured the gene expression of the mitochondrial thermogenic marker, Uncoupling protein 1 (Ucp-1). Intriguingly, we observed a higher expression of Ucp-1 in old mice compared to adult (p = 0.006) (Fig. 4B). As previously observed in humans^36^, the Ucp-1 gene expression varied greatly between individuals and we detected no effect of the exercise training intervention.

## Discussion

We here describe an age-dependent phenotype of visceral adipose tissue of exercising mice and report an accumulation of alternatively activated (CD206+) M2 macrophages in old mice in response to endurance training. This phenotype was accompanied by less visceral fat, smaller adipocytes, as well as higher Ucp-1 and IL-10 mRNA expression while Tgf-β1 mRNA expression was lower compared to the younger counterparts.

When interpreting our data in the light of the literature, it is important to bear in mind that we utilized a model of very old (23 months) mice, which we compared to adult mice. This could explain why our results conflicted with previous reports on increased visceral fat in old mice^12,13,37^, as a bimodal pattern with decreased visceral fat has been observed^38–40^. In accordance with our study, Donato and colleagues found that 30 months old (ancient) mice exhibited less visceral fat and smaller adipocytes compared to adult mice (6 months)^38^. However, in sharp contrast to our findings, that study reported a decrease in Ucp-1 and an increase in fibrosis, while we demonstrate the opposite phenotype.

Certainly, the present study has limitations. For one, variation in rest before sacrifice ranged from 3–5 hours thus introducing possible spatial variation in the conducted gene-expression measurements. Hence, our measurements do not reflect acute work/training response, but rather a habitual adaptation. Further, we are aware that CD206 is not a marker exclusively reserved for alternatively activated macrophages^41^, but is a generally accepted M2 marker. Importantly, a previous study applying the M2 macrophage markers CD163 and Mrc1, support our findings of an increase in M2 macrophages in mice with ageing, although in this study, mice were only aged for 30 weeks^42^. Furthermore, the fact that no circulating blood samples were available, limits the ability to conclude regarding the coupling between local adipose tissue changes and alterations in circulating levels of inflammatory markers.

Nevertheless, we here describe an anti-inflammatory phenotype of visceral adipose tissue in old mice, whereas ageing (and obesity) -induced changes in adipose tissue is originally presumed to be based upon an increasingly inflammatory, and not anti-inflammatory, skewing^43,44^. Therefore, our data represent an important contribution to the literature, indicating that (pronounced) aging per se, does not generate a pro-inflammatory phenotype or visceral fat accumulation in mice. Interestingly, a cross sectional study on human ageing found that from around the 8^th^ to the 9^th^ decade, a reduction in waist circumference (surrogate marker for abdominal obesity) was observed^45^, supporting the notion of decreased visceral fat with pronounced ageing. However, whether the immunological phenotype of visceral adipose tissue in very old humans is indeed dominated by anti-inflammatory processes as our data would suggest, remains unanswered.

In our study, the visceral adipose tissue of the old mice seemed either more lipolytic or had lost lipid storage capacity. This was observed at rest and was accentuated following exercise training as epidydimal fat mass was reduced in combination with smaller adipocytes (Fig. 1A–C). The interaction between adipose tissue macrophages and lipolysis has been previously discussed^27^, and it has been shown that local lipid fluxes is a potent mediator of macrophage recruitment to adipose tissue^26^.

Indeed, exercise is a powerful mediator of lipolysis, and it has been shown *in vivo* in humans that the lipolytic activity is higher in abdominal depot (represented by both visceral and subcutaneous adipose tissue) than in the gluteal depot (consisting of subcutaneous adipose tissue)^46^. This is in line with our finding in which ET decreased visceral adipocyte size in both age groups, a phenomenon already touched upon by other researchers^47,48^. Moreover, our finding that only ET seemed to reduce visceral fat mass is consistent with one of the few available meta-analysis on the subject^23^. Interestingly, in concordance with our observations, exercise has previously been established to generate an anti-inflammatory response, by increasing the expression and release of anti-inflammatory mediators such as IL-10, arginase-1 and IL-6 (acute release without TNF-α) from human leukocytes and skeletal muscles^49,50^. It has also been suggested that exercise might confer a shift from M1 to M2 macrophage phenotype in adipose tissue^51,52^.

As endurance training promote browning of white adipose tissue in mice^20,35^, it is interesting to note that pre-adipocytes obtained from epididymal fat tissue have the ability to acquire a brown-like phenotype regulated by PPAR-γ, PGC1-α and norepinephrine, which are all known to be involved in response to exercise^53^. Interestingly, some studies advocate that M2 macrophages, through norepinephrine release, can increase UCP-1 expression and mediate browning of white adipose tissue^21,54^, which was later questioned by other groups, discarding this idea^55^. Here, we report a modestly elevated expression of UCP-1 (Fig. 4B) in old mice, while the mechanism or direct link to the higher amount of M2 macrophages could not be determined within the scope of the current study. Importantly, our results support an increased oxidative phenotype of VAT generated by exercise, as PGC1-α expression was increased (Fig. 4A). This is consistent with existing literature where PGC1-α is seen upregulated in both muscle and adipose tissue following an intense exercise protocol^56^, supporting the concept that exercise training can convey a beneficial metabolically effect on visceral adipose tissue^57^.

In conclusion, our study emphasizes the dynamics of adipose tissue and describe the visceral adipose tissue of lean old mice as an anti-inflammatory and highly lipolytic tissue with endurance exercise further enhancing these characteristics.

## Supplementary information

Supplementary data

## Data Availability

All data are freely available upon request.
