# Supplementary material for: An anti-inflammatory phenotype in visceral adipose tissue of old lean mice, augmented by exercise
Source: Sci Rep. 2019 Aug 19;9:12069. doi: 10.1038/s41598-019-48587-2 (PMC6700172; doi:10.1038/s41598-019-48587-2)
Supplement: Supplementary file 1 — Supplementary data [file 41598_2019_48587_MOESM1_ESM.pdf]

Supplementary data for:

An anti-inflammatory phenotype in visceral adipose tissue of old lean mice, augmented by exercise

*+Ziegler A. K.[1,2], +Damgaard A.[1], Mackey A. L. [1,2], Schjerling P.[1], Magnusson P. [1, 3], Olesen A. T. [1], Kjaer M.[1,2], Scheele C.[4,5]

[1]: Institute of Sports Medicine Copenhagen, Department of Orthopedic Surgery M, Bispebjerg Hospital and Center for Healthy Aging, Faculty of Health and Medical Sciences, University of Copenhagen, Denmark. [2]: Center for Healthy Aging, Department of Biomedical Sciences, Faculty of Healthy and Medical Sciences, University of Copenhagen, Denmark.[3]: Department of Physical Therapy, Musculoskeletal Rehabilitation Research Unit, Bispebjerg Hospital, Denmark. [4]: The Centre of Inflammation and Metabolism and Centre for Physical Activity Research Rigshospitalet, University Hospital of Copenhagen, Denmark, [5]Novo Nordisk Foundation Center for Basic Metabolic Research, Faculty of Health and Medical Sciences, University of Copenhagen, Denmark

*Corresponding author: andreas.kraag.ziegler.01@regionh.dk

+Authors contributed equally to the study

Figure S1. Schematic depiction of exercise protocol for RT and ET

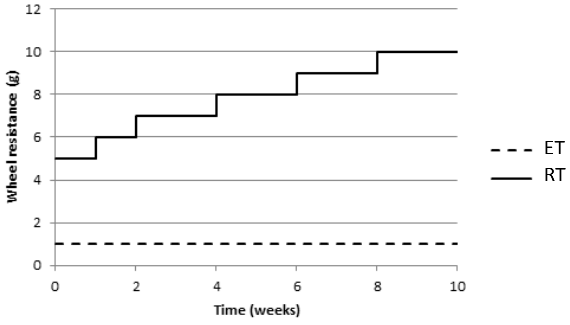

**Table S1,** primers for gene-expression in the mice study			
**Gene**	**Transcript**	**Forward Primer**	**Reverse Primer**
**GAPDH**	NM_001289726.1	AACTTTGGCATTGTGGAAGG	GGATGCAGGGATGATGTTCT
**UCP-1**	NM_009463.3	GGCCTCTACGACTCAGTCCA	TAAGCCGGCTGAGATCTTGT
**PGC-1α**	NM_008904.2	CCCATACACAACCGCAGTC	GAACCCTTGGGGTCATTTG
**IL6**	NM_031168.2	AAGTGCATCATCGTTGTTCATACA	GAGGATACCACTCCCAACAGACC
**IL10**	NM_010548.2	CCAGTACAGCCGGGAAGACA	AGTCCGCAGCTCTAGGAGCAT
**Adiponectin**	NM_009605.5	GGAGAGAAAGGAGATGCAGGTC	CCCCGTGGCCCTTCA
**TNF-α**	NM_013693.3	ATGGCCTCCCTCTCATCAGT	TTTGCTACGACGTGGGCTAC
**TGF-β1**	NM_011577.2	AGGGCTACCATGCCAACTTC	CCACGTAGTAGACGATGGGC
